# Growth and Structure of Ultrathin Iron Silicate and Iron Germanate Films

**DOI:** 10.1021/acs.jpcc.4c05601

**Published:** 2024-10-31

**Authors:** Gina Peschel, Alexander Fuhrich, Dietrich Menzel, Mirali Jahangirzadeh Varjovi, Sergio Tosoni, Hans-Joachim Freund

**Affiliations:** † Fritz Haber Institute of the Max Planck Society, Faradayweg 4-6, Berlin D-14195, Germany; ‡ Physics-Department E20, Technical University of Munich, Garching b. München 85748, Germany; § Department of Materials Science, University of Milano-Bicocca, Via Roberto Cozzi 55, Milano 20125, Italy

## Abstract

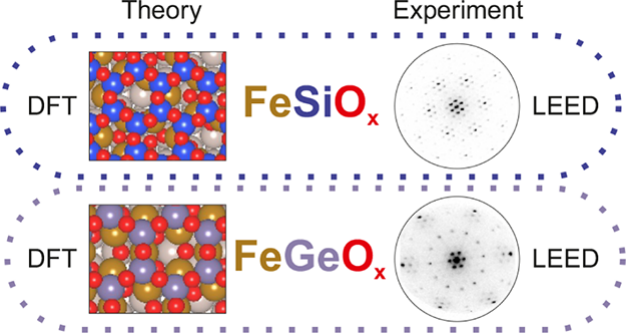

The growth and structure of two-dimensional iron silicate and iron germanate films on Ru(0001) are studied. We investigate in detail the temperature-dependent film formation of ultrathin layers of iron silicate and iron germanate. These two-dimensional films can be seen as model systems for more complex catalytically active structures, such as zeolites, which can be used as selective catalysts or molecular sieves. The experimental methods of XPS, LEED, LEEM, LEEM-IV, and XPEEM are applied for correlated chemical and physical characterization in situ and in real time, and DFT is applied for theoretical consideration. We show that both systems can be considered as two-layered systems, with a monolayer of iron oxide at the Ru interface and a monolayer of silica or germania on top, respectively. The Fe-Fe distance in the iron oxide layer is influenced by the Si-O-Si or Ge-O-Ge bond length, in agreement with those of unstrained silicates or germanates. Moreover, iron silicate can be prepared using different preparation methods. The actual loading of Fe atoms is three per unit cell for FeGeO_x_ and only two for FeSiO_x_.

## 1 Introduction

Two dimensional silica layers have been investigated extensively since they have proved great suitability to study structure and reactivity relations as model systems for catalysts.^1^ Ultrathin silica films can be prepared on various substrates like Ru(0001),^2^ Pt(111),^3^ Pd(111),^4^ or Au(111).^5^ While on Mo(112) only the crystalline monolayer is formed, on Ru(0001) both the monolayer, the crystalline and vitreous bilayer as well as the zigzag structure of silica can be observed.^6,7^ The metal support plays a major role in the silica and also in silicate films. The silica monolayer is strongly bound to the Ru(0001) substrate, while the bilayer lifts up and binds to the substrate only via van der Waals forces.

The silica monolayer (ML) and the germania monolayer on Ru(0001) comprise corner-sharing SiO_4_ and GeO_4_ tetrahedra, respectively. Both silica and germania monolayers consist of 6-fold-coordinated, in plane ring systems which are linked via oxygen atoms to Ru(0001) and hence chemically bound to the substrate. In contrast to the ultrathin silica monolayer, the germania monolayer exhibits more variations in the angular arrangement of the tetrahedral building^8^ units and a more graded interaction of the film system with Ru(0001).^9^ The germania monolayer, the bilayer, and the zigzag polymorph can be prepared on Pt(111), similar to the Ru(0001) support for the silica system.^10^ The different interaction with the metal substrates leads to different silica or germania structures.^10^ Furthermore, the chemical stability of silica films on Pt(111) and Rh(111) is observed to be different.^11^ In addition to pure silica or germania films, also mixed germania-silica films have been prepared on Ru(0001).^12^ Due to the more structural flexibility of the Ge-O-Ge bonds in comparison to Si-O-Si bonds, mixed germania-silica films offer opportunities for membranes.

By the incorporation of metal atoms such as aluminum, titanium, and iron, the chemical reactivity of the silica and germania films can be changed. The aim is a model system for zeolites, which are known for their high catalytic activity and therefore are used in many industrial applications. When aluminum is added to silica preparation, it can be found homogeneously distributed over the film.^13^ In fact, Al^3+^ atoms are found to substitute Si^4+^ atoms in the upper and lower levels of a silica bilayer. Apart from that, the silica structure is unchanged. The minimal distance of the Al atoms in this matrix obeys Löwenstein’s rule.^14^ However, iron and titanium are proven to incorporate differently into the silica matrix. Theoretical models show that iron is only found in the lower level of the silica bilayer.^15^ Experiments show that iron is not simply substituting silicon atoms, but a two-layered system is formed. In contact to the Ru(0001) substrate, a layer of iron oxide forms, similar to FeO on Ru. On top of this iron oxide layer, a monolayer of silica exists, which is rotated by 30°. The silicon atoms are bound through oxygen to the iron atoms underneath.^16^ The composition of iron silicate on Ru(0001) and Pd(111) is Fe_2_Si_2_O_9_, where one oxygen atom is bound to the support.^15,17^ The same structure exists, when iron is exchanged by titanium.^18^ First studies to incorporate aluminum and iron together have been presented.^19^ However, in case of low amounts of material, aluminum and iron cannot be found in the same domain.

Iron silicate and iron germanate are prepared in a stepwise preparation on the basis of a well-prepared monolayer or bilayer of FeO. The FeO layers are prepared by direct deposition at elevated temperatures. Palacio et al.^20^ showed that the background pressure determines whether a monolayer or bilayer is grown. However, the chemical nature of the monolayer and bilayer was not evaluated by them. It will be shown that the bilayer contains an additional oxygen layer at the Fe/Ru interface, while this is not the case for the monolayer of FeO. We will show that iron silicate can grow on both a FeO monolayer and a bilayer. The silica layer orders in a similar manner on both films. By using incomplete layers, we will show that iron silicate is energetically favored on a single iron oxide layer and that the iron oxide layer has a lower iron concentration than the monolayer of FeO.

The addition of iron to silica films was studied by Włodarczyk et al.^15^ IRAS measurements and DFT calculations indicate a vertical separation of a silica layer and an iron oxide layer, instead of intermixing of iron and silicon. Up to now, only the final structure of these ultrathin iron silicate films has been reported.^15,19,21^ The formation process, temperature dependencies, and the precise structure remain unknown. Based on the knowledge of ultrathin germania layers on Ru(0001), the next step is to substitute silicon in iron silicate films by germanium. Due to similar properties and bond lengths, this is expected to be possible.

In this work, we have studied the formation process of ultrathin layers of iron silicate (FeSiO_x_) and iron germanate (FeGeO_x_) in detail by spectro-microscopy and DFT calculations. We show that FeSiO_x_ can be prepared based on either a disordered or an ordered silica monolayer. In both cases, the final films show the same characteristics. Furthermore, we show that Ge and Si behave similarly in the combination with iron oxide.

## 2 Experimental Details

The measurements have been performed at the spectro-microscope SMART,^22^ operating at the high flux beamline UE49PGM at the synchrotron facility BESSY II of the Helmholtz Center for Materials and Energy in Berlin (HZB). This made it possible to obtain and correlate in situ and in real time the results of XPS, LEED, LEEM, LEEM-IV, and XPEEM for stages of preparation and evolution of well-defined samples. The Ru(0001) single crystal was mounted on a commercial ELMITEC sample holder, fixed by a Mo cap, and heated from the back either by radiation for temperatures up to 700 K or by electron bombardment for higher temperatures. Temperatures were measured with a W26%Re/W5%Re thermocouple spot-welded to the sample support and additionally with a pyrometer for temperatures above 520 K. The materials were deposited using commercial Focus EFM3 evaporators. Iron was evaporated from a rod (purity 99.99%) and germanium from a crucible (purity 99.999%). The Ru(0001) single crystal was cleaned by several Ar^+^ sputtering and annealing cycles before iron, germanium, or silicon was deposited. Cleaning was performed in three steps: First, Ru(0001) was oxidized at 1170 K for 10 min in 1.0·10^–6^ mbar of oxygen; second, the oxygen was pumped down, and subsequently, the sample was annealed at 1420 K in UHV for 10 min; and finally, the sample was flashed to 1520 K in UHV conditions for only 1 min. All film preparations were performed in situ and followed in real-time by low energy electron diffraction (LEED). The following preparation recipes optimized by exploratory work were applied for film formation.

Stepwise recipe for iron silicate: FeO on Ru(0001) can be prepared by direct deposition of iron at 800 K in an oxygen pressure. The oxygen pressure itself has a huge impact on the resulting film thickness.^20^ An oxygen pressure of 2.0·10^–8^ mbar leads to the formation of a monolayer of FeO, while a pressure of 1.0·10^–7^ mbar O_2_ leads to the formation of a bilayer of FeO. Silicon is deposited in 2.0 10^–7^ mbar O_2_, subsequently. The silicon amount used equals the amount necessary to form a monolayer of silica. Finally, the film was oxidized in 1.0 10^–6^ mbar and first at RT and then stepwise up to 660 K, 800 K, 900 K, and 1000 K. The sample was oxidized at 660 K for 30 min and for higher temperatures for 15 min at the individual temperatures. After each temperature step, the film was cooled down to RT without applying further oxygen and analyzed with LEED, LEEM-IV, and XPS.

The same iron silicate characteristics in LEEM-IV, LEED, and XPS are achieved by using a so-called combined recipe. The recipe is described in the following.

Combined recipe for iron-silicate: In this preparation, the Ru(0001) substrate is oxygen precovered with a 3O-(2 × 2) adlayer.^6^ This layer is achieved by oxidation of the freshly cleaned Ru(0001) substrate in 1.0·10^–6^ mbar at 1170 K with subsequent cooling to room temperature (RT) in the same oxygen pressure. A freshly cleaned Ru(0001) substrate is used as support. Silicon and iron were deposited subsequently in 2.0·10^–7^ mbar oxygen pressure at RT. Afterward, the films were oxidized up to 1000 and 1080 K in 1.0·10^–6^ mbar of oxygen on Ru(0001).

Stepwise recipe for iron germanate: First, a monolayer of O_2_ was prepared, following the method described in.^6^ Then, an incomplete layer of GeO_2_ was produced. To prepare GeO_2_, a thin film of germanium was deposited at 540 K in UHV on clean Ru(0001). The deposition was stopped before the complete layer was closed. Afterward, this film was oxidized in 1.0·10^–6^ mbar of oxygen at a temperature of 670 K for 10 min. The resulting film was cooled down to RT in 1.0·10^–6^ mbar oxygen. This leads to germanium-rich areas, while others are germanium-free. Subsequently, an ML of iron was deposited at RT on this GeO_2_ layer in 2.0·10^–7^ mbar of oxygen. Finally, the film was oxidized in 1.0·10^–6^ mbar up to 890 K. Before the next oxidation step took place, the film was cooled down to RT and analyzed at this temperature. While this procedure was followed in the majority of characterizing spectra, the ability of the system to take spectra while heating the sample or keeping it at elevated temperatures was also used to pinpoint characteristic processes, e.g., to follow disorder transitions by LEED, or film growth and local redistribution processes by LEEM images.

The iron amount was calibrated by direct deposition at 620 K in 2.0·10^–8^ mbar oxygen pressure. Here, a single FeO layer growth was observed that allowed estimating the necessary time to complete the monolayer.

## 3 Computational Details

All calculations were done with the GPU-accelerated version of code VASP 6^23−26^ within the framework of spin-polarized density functional theory (DFT).^27,28^ The exchange-correlation potential was parametrized using the Perdew–Burke–Ernzerhof (PBE)^29^ functional within the generalized gradient approximation (GGA). The projector-augmented wave (PAW)^30^ approach was utilized to treat the core-valence interaction. To expand the electronic wave function, a plane-wave basis set with a kinetic cutoff energy of 520 eV was utilized and a vacuum layer of ∼ 20 Å was included in the nonperiodic dimension of all slab model calculations to hinder the unrealistic interactions among adjacent images. The electronic relaxation convergence threshold between consecutive steps in total energy calculations was less than 10^–5^ eV. The truncation criterion for structural optimization (ionic loops and lattice constants) was set to 0.01 eV/Å. Brillouin zone sampling was performed in the Γ-point only, as justified by the large dimension of the supercell (vide infra). To determine the net charge transfer between the oxide and silicate films and their support, the Bader technique was utilized.^31^ Additionally, long-range dispersion effects were taken into account based on the D3 approach from Grimme, which incorporates the Becke-Johnson damping function.^32,33^ A Hubbard parameter of 3 eV was applied to the 3d-orbitals of Fe to account for the strongly correlated nature of these electrons.^34^ This setting was previously tested and discussed to study the growth of iron oxide films on platinum.^35^

## 4 Results and Discussion

### 4.1 Initial FeO Layer

**Figure 1:**
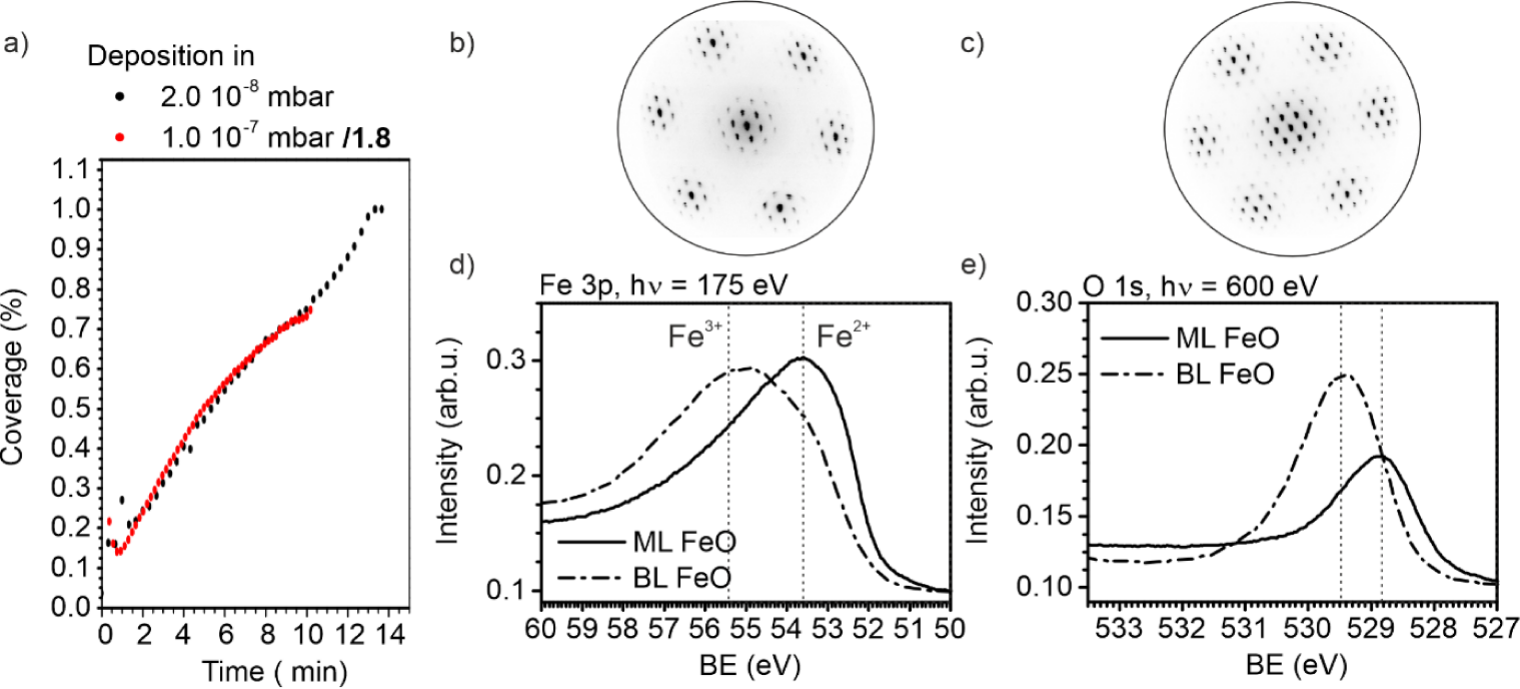
Preparation of FeO by using an oxygen pressure of 2.0·10^–8^ mbar for a FeO monolayer and 1.0·10^–7^ mbar O_2_ for a FeO bilayer on Ru(0001) by direct deposition at 800 K. (a) FeO coverage observed by LEEM during deposition. (b) LEED pattern (42 eV) of a completely closed FeO monolayer. (c) LEED pattern (42 eV) of a completely closed FeO bilayer. (d,e) XPS measurements of a freshly prepared FeO monolayer and bilayer on Ru(0001) for the Fe 3p line (d) and O 1s line (e).

In this work, iron silicate is prepared by silicon deposition on a pure FeO layer of monolayer or bilayer height. In order to evaluate the influence of the FeO layers on the iron silicate films, in this subsection, the FeO mono- and bilayer films are discussed. The FeO films begin to grow on the step edges, as can be followed in LEEM. The film coverage increases with deposition time until a complete FeO layer is formed. The LEEM images in Figure 1a show the coverage increase for an oxygen pressure of 2.0·10^–8^ mbar to form a monolayer of FeO and 1.0·10^–7^ mbar O_2_ to form a bilayer of FeO with the same evaporator settings. At constant temperature, the FeO growth rate decreases by a factor 1.8 when the iron deposition takes place at 1.0·10^–7^ mbar O_2_ instead of 2.0·10^–8^ mbar O_2_. Further evidence of the varying FeO film thickness caused by different oxygen pressures is found by STM.^20^ The resulting LEED structures of FeO prepared at 2.0·10^–8^ mbar and 1.0·10^–7^ mbar are shown in Figure 1b,c, respectively. Both films contain the characteristic LEED pattern of FeO,^20^ namely a Moiré pattern with “6 on 7” reconstruction. Since a Moiré structure results from the superposition of two lattices with similar unit cells, the “6 on 7” reconstruction indicates that six iron atoms fit commensurable on seven ruthenium atoms. Moreover, the FeO bilayer contains higher orders (Figure 1c). Lower pressure than 2.0·10^–8^ mbar leads to individual domains rotated by ± 17° (Wood notation: (1.15 × 1.15)R17° and (1.15 × 1.15)R163°). The rotated structures have a 1.15 times larger unit cell than the Ru(0001) substrate. For intermediate pressures between 2.0·10^–8^ mbar and 1.0·10^–7^ mbar, a mixture of monolayer and bilayer domains grows next to each other.

FeO typically consists of a stack of alternating layers of Fe^2+^ cations and O^2–^ anions, both arranged in a hexagonal lattice form.^35^ The XPS Fe 3p and O 1s lines of the just grown films are depicted in Figure 1d,e, respectively. The FeO monolayer film grown in 2.0·10^–8^ mbar fulfills the expectation by containing only a Fe^2+^ component at 53.6 eV with the corresponding O 1s line at 528.9 eV. In contrast, in the FeO bilayer grown in 1.0·10^–7^ mbar, two main features are detected: the Fe^2+^ component (binding energy of 53.6 eV) and a further component at a binding energy of 55.6 eV attributed to Fe^3+^can be identified. Moreover, the O 1s line shows an additional component at 529.6 eV. Since the LEED pattern equals that of FeO layers, the hexagonal stacking of alternating iron and oxygen layers is very likely also present in the bilayer FeO films. Thus, the Fe^3+^ component indicates the presence of an additional oxygen layer: Ru/O/Fe/O/Fe/O.

### 4.2 Complete Layers of Iron Silicate

Iron silicate is prepared by using a complete layer of a monolayer or bilayer of FeO. The resulting films will be addressed as ML-FeSiO_x_ and BL-FeSiO_x_, respectively. The characteristics of the monolayer and bilayer of FeO have been described in the previous section.

**Figure 2:**
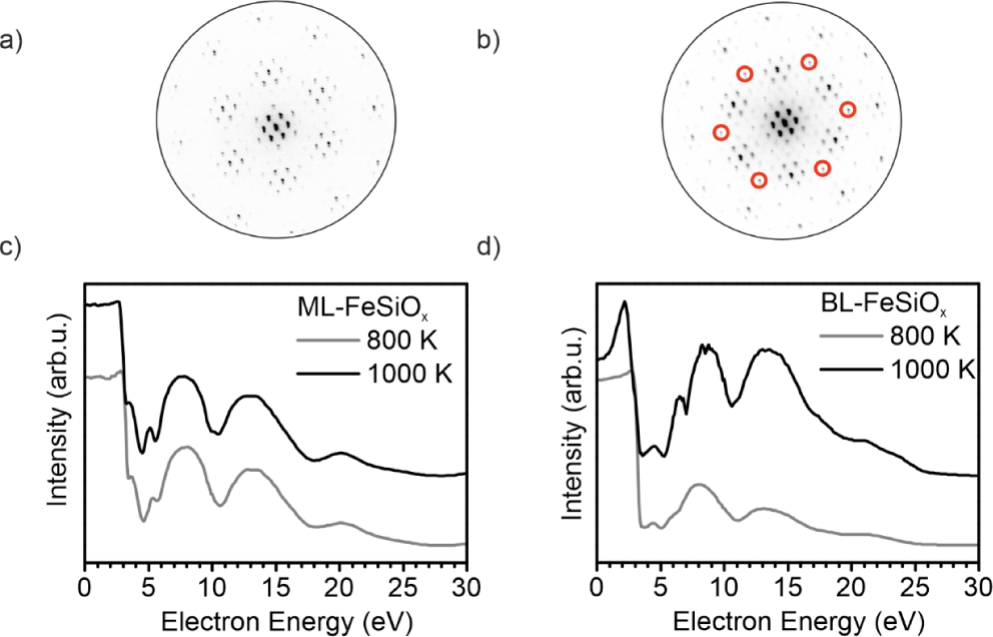
LEED and LEEM-IV comparison of iron silicate based on a FeO monolayer or bilayer. (a) LEED pattern (42 eV) of a ML-FeSiO_x_ film after oxidation at 1000 K in 1.0·10^–6^ mbar. (b) LEED pattern (42 eV) of a BL-FeSiO_x_ film after oxidation at 1000 K in 1.0·10^–6^ mbar with highlighted (3 × 3) LEED spots. (c,d) LEEM-IV curves of the iron silicate films after oxidation at 800 K (intermediate temperature step) and 1000 K (final temperature step). (c) ML-FeSiO_x_ and (d) BL-FeSiO_x_.

In Figure 2a,b, the LEED structures of the ML-FeSiO_x_ and BL-FeSiO_x_, respectively, are given. The final oxidation temperature is 1000 K. Both films give rise to nearly the same LEED structure, namely a Moiré pattern with “8 on 9” reconstruction surrounding the (00) spot and additional spots rotated by 30° with respect to the high symmetry directions of Ru(0001). Additionally, the BL-FeSiO_x_ film contains (3 × 3) LEED spots, which are highlighted in the LEED pattern (Figure 2b).

The corresponding LEEM-IV curves are depicted in Figure 2c,d for the ML-FeSiO_x_ and BL-FeSiO_x_, respectively. A LEEM-IV curve can be used as a fingerprint for a specific system and will be used in this work accordingly. The LEEM-IV curves were measured at RT for films annealed at 800 K (intermediate temperature) and 1000 K (final annealing temperature). The MEM-LEEM border of ML-FeSiO_x_ is found at 3.08 eV. This value is correlated to the workfunction of the film by a correction factor of 3.2 eV to be 6.28 eV. The difference between the curves after 800 and 1000 K for the ML-FeSiO_x_ film is mainly found in the energetic range closely above the MEM-LEEM border up to 7 eV. After 800 K, mainly one dip at 4.5 eV is present, while after 1000 K, two dips at 4.5 and 5.5 eV are found. The development of the second peak at 5.5 eV can be used as a quality factor of the film. For energies above 7 eV, both films give rise to three main peaks at 7.5 eV, 13 eV, and 20.4 eV.

The LEEM-IV curve of the BL-FeSiOx film clearly differs (see Figure 2d). The MEM-LEEM border is found at 2.79 eV (workfunction: 5.99 eV). The difference to the ML-FeSiO_x_ film indicates a lower surface dipole in the BL-FeSiO_x_ film. Similar to the ML-FeSiO_x_ raising the temperature from 800 to 1000 K is mainly influencing the energetic range close to the MEM-LEEM border. After 800 K, two dips are present at 3.8 and 5.1 eV, while after 1000 K, an additional peak at 7.0 eV develops. The dip at 7 eV of the LEEM-IV curve can be used as an indicator for film quality. For higher energies, the same peaks at 8 eV, 13 eV, and 21.1 eV are found, however, with different peak intensities.

**Figure 3:**
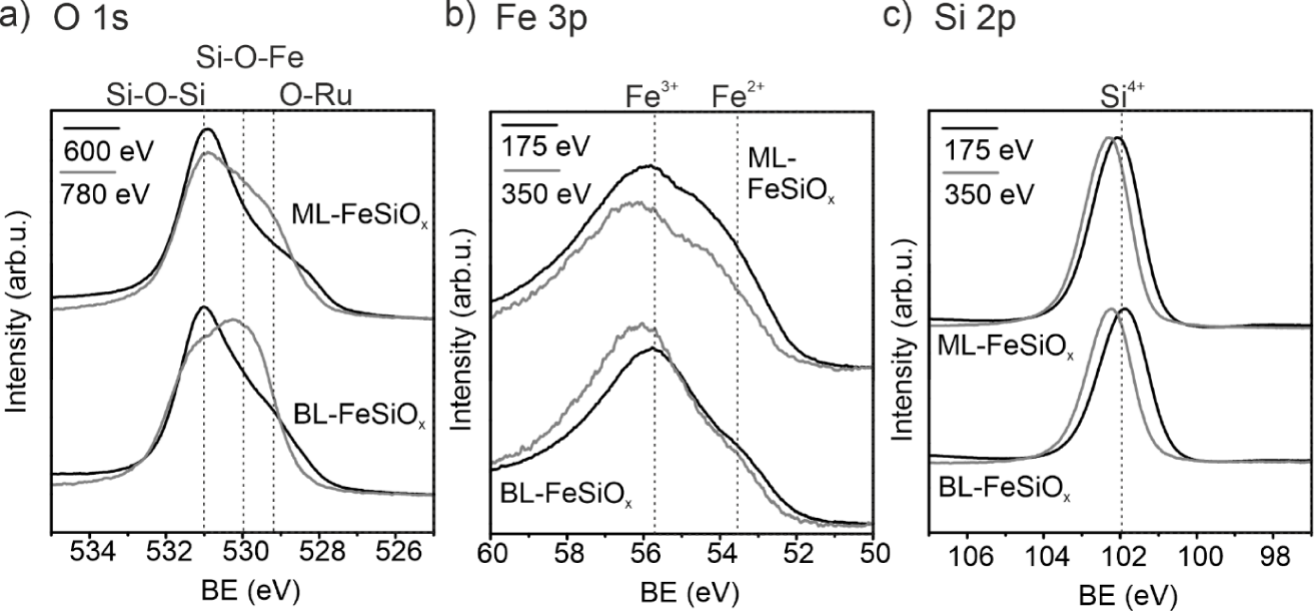
XPS spectra for ML-FeSiO_x_ and BL-FeSiO_x_ after oxidation at 1000 K in 1.0·10^–6^ mbar O_2_. Measurements with varying photon energy are given in black (surface-sensitive) and gray (less surface-sensitive). (a) O 1s line, (b) Fe 3p line, and (c) Si 2p line.

The XPS line for the final ML-FeSiO_x_ and BL-FeSiO_x_ films (oxidation temperature: 1000 K) is given in Figures 3a–c for the O 1s, Fe 3p, and Si 2p lines, respectively. In order to gain information about the film stacking, two different energies with different free mean paths of the electrons are chosen. One of the energies is surface-sensitive with kinetic energies of 70 eV (O 1s, Si 2p) or 120 eV (Fe 3p) (black curve), and the other is less surface-sensitive with kinetic energies of 250 eV (O 1s, Si 2p) or 305 eV (Fe 3p) (gray curve). The Fe 3p line has in both cases two components: Fe^2+^ and Fe^3+^. While for both films, the Fe^3+^ component is more pronounced, and the intensity ratio I(Fe^3+^):I(Fe^2+^) is larger in BL-FeSiO_x_ than in ML-FeSiO_x_. Moreover, the depth profile indicates that the I(Fe^3+^):I(Fe^2+^) ratio in BL-FeSiO_x_ is even larger for higher kinetic energies. This indicates that iron atoms with Fe^3+^ configuration are found closer to the ruthenium substrate. In case of ML-FeSiO_x_, no difference in the depth profile is found. The reason is the presence of only one iron oxide layer. As indicated in the Si 2p line (Figure 3c), silicon is completely oxidized in the Si^4+^ state. This is the case for iron silicate preparations with both FeO thicknesses.

The XPS O 1s line (Figure 3a) contains three components at 529.3 eV, 530.0 eV, and 531.0 eV. For comparison, a crystalline SiO_2_ ML on Ru(0001) contains two components in the O 1s core level at 529.1 and 530.7 eV binding energy. Thus, the component at 531.0 eV can be assigned to Si-O-Si bonds, and the other to O-Ru. The contribution of the single components of the O 1s core level is shown in Figure S1. Comparing the energetic positions with the O 1s line of FeO (Figure 1e) at 528.9 eV (ML FeO) or 529.4 eV (BL FeO) indicates a Fe-O-Fe component overlapping with the O-Ru component. The component at 530.0 eV is energetically between these two components and can therefore be assigned to Si-O-Fe (see also^15^). For iron silicate preparations with both FeO thicknesses, the ratio I(Si-O-Si):I(Fe-O-Fe/O-Ru) is relatively large, when measured with surface-sensitive kinetic energies (*E*_kin_ = 70 eV, hν = 600 eV). On the other hand, kinetic energies with higher free mean paths of the electron (*E*_kin_ = 250 eV, hν = 780 eV) reduce the ratio strongly in case of ML-FeSiO_x_ and reverse for BL-FeSiO_x_.

The depth profile results confirm the two-layered nature of the films. In both cases, a monolayer of silica is formed on top of the FeO layers. Thus, silicon does not diffuse into the initial FeO layers. The silica layer is rotated by 30°, and the Si-O-Si bond length corresponds to the Fe-Fe distance of the iron oxide layer. As indicated by the Fe-O-Si component, the two layers are interconnected by oxygen bonds. Moreover, the second FeO layer is still present in BL-FeSiO_x_ as deduced by the different Fe-O-Fe concentrations considering the identical silicon amount. During the oxidation process, the original “6 on 7” reconstruction of the pristine FeO layers (Fe-Fe distance: 3.16 Å) is altered to a “8 on 9” reconstruction (Fe-Fe distance: 3.04 Å) and thus a smaller iron distance. Both iron oxide layers in BL-FeSiO adapt to Fe-Fe distance reduction. The silica layer appears to be identical for both iron silicate preparations independent of the number of FeO layers. However, the additional (3 × 3) structure indicates a corrugation of the BL-FeSiO_x_ layer in every second silica 6-fold ring.

For the complete iron oxide layer, it cannot be determined from the experimental results whether the iron concentration remains the same as in FeO or whether it is altered during oxidation. In fact, the same LEED spectra could be achieved with one less iron atom per silica unit cell.^16^ In this case, the missing atoms in the iron oxide layer would also give rise to the 30° rotated spots. This question is further addressed in the discussion of incomplete layers of iron silicate.

### 4.3 Incomplete Films ML-FeSiO_x_

**Figure 4:**
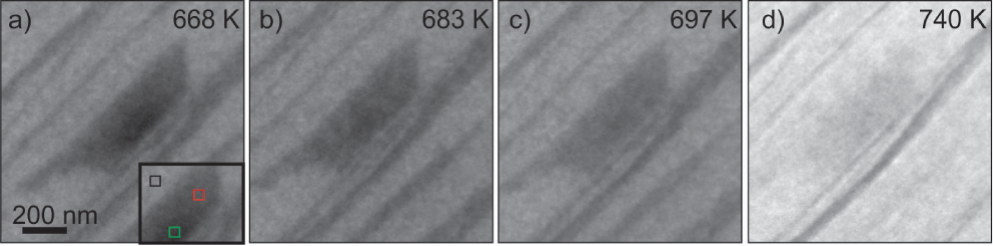
In situ oxidation process of an incomplete layer of ML-FeSiO_x_ with 87% of FeO coverage observed in LEEM. We distinguish the various domains as type α (FeO-Si/Ru, like black square in (a)) and type β (like red and green square in (a)). LEEM images are taken at 15 eV during oxidation in 1.01.0*10·10^–6^ mbar O_2_ at the indicated temperatures. The average heating rate is 0.15 K/s.

The advantage of incomplete layers of iron silicate is the possibility to follow dynamical processes driven by concentration change in the material. The incomplete layers are prepared by using unclosed FeO monolayers (FeO concentration < 100%) and silicon necessary to form complete layers of SiO_2_. Thus, two domains with different material compositions are present: domains of type α (FeO + Si/Ru) and type β (Si/Ru). The films are oxidized in 1.0·10^–6^ mbar with increasing temperature, and the process is followed in LEEM. An example of the oxidation process is given in Figure 4. Domain α is indicated by a black square. Domain β is indicated in red and green. During oxidation, the reflectivity in domain α increases homogenously (compare Figure 4a,d). In contrast to this, in domain β, an inhomogeneous change of reflectivity and roughness is found. The transformation takes place as a front starting at domain α and moving toward the center of domain β. In Figure 4a, half of domain β is already transformed (indicated in red). The initial contrast is still present in the area indicated in green. In Figure 4b, the complete domain β is transformed. For higher temperatures or oxidation time, the reflectivity of domain β increases and the intensity contrast between domains of type α and type β diminishes (Figure 4d).

**Figure 5:**
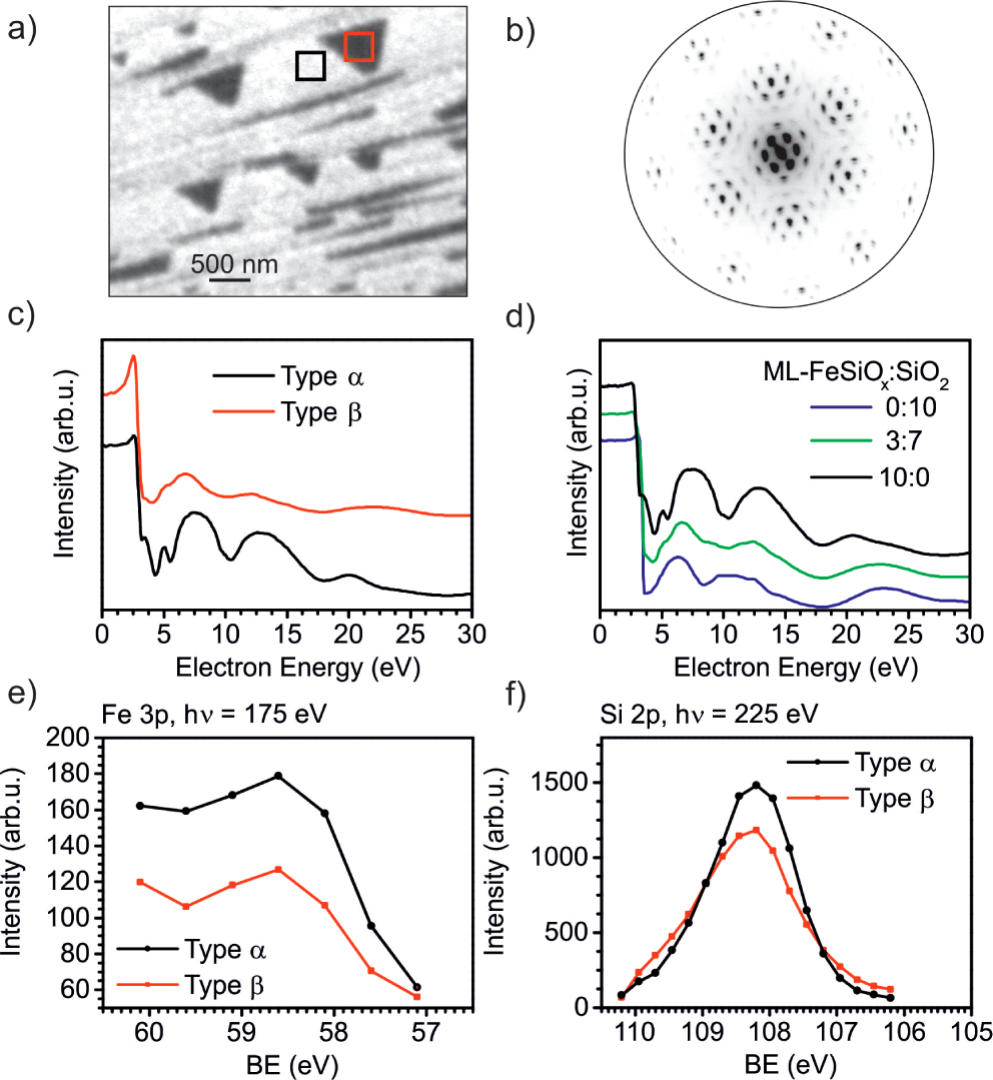
Characteristics of iron silicate based on an incomplete layer of monolayer-thick FeO. The results are based on a FeO coverage of 87%. (a) LEEM image at 17.2 eV and (b) LEED at 42 eV. (c,d) LEEM-IV curves of the measured domains (c) and comparison curves (d). For the comparison curves, the LEEM-IV curve of a complete layer of ML-FeSiO_x_ (see Figure 2c) and of a complete layer of a monolayer of SiO_2_ is given. The green curve is a linear combination with a ratio of 3:7 of the FeSiO_x_ and SiO_2_ curves. (e,f) XPEEM data taken in neighboring domains of type α and β. (e) Fe 3p line and (f) Si 2p line. The Fe 3p and Si 2p lines are not taken at the same spot.

In Figure 5a,b, the LEEM-IV and the μLEED pattern of the final film (oxidation at 1000 K) are given. The LEEM image indicates different contrast in domain α and domain β. The μLEED pattern is taken of a 17 μm^2^ area containing both domains. However, the LEED pattern shows only the presence of one structure: a Moiré structure with “8 on 9” reconstruction and by 30° rotated spots. The μLEED pattern in Figure 5b is similar to the LEED pattern of the completely closed ML-FeSiO_x_ film shown in Figure 2a. The measured LEEM-IV fingerprints of domains of type α (black) and type β (red) are depicted in Figure 5c. Domain α contains the same fingerprint as complete layers of ML-FeSiO_x_ and can thus be considered as identical (compare Figure 2c) in structure and composition. The well-developed dip at 5.5 eV indicates a comparable quality of the film as in complete layers. The LEEM-IV curve of domain β matches neither the fingerprint of ML-FeSiO_x_ nor the fingerprint of ML-FeSiO_x_ and the fingerprint of a monolayer of SiO_2_/Ru (Figure 5d in violet). However, the measured curve can be produced by linear combination of normalized curves of ML-FeSiO_x_ and ML SiO_2_ with (0.3·I_ML-FeSiOx_ (*E*_kin_) + 0.7·I_ML SiO2_(*E*_kin_)) /(I_ML-FeSiOx_ (*E*_kin_) + I_ML SiO2_(*E*_kin_)). Here, the size of individual areas with ML-FeSiO_x_ and ML SiO_2_ is considered large enough to have noninterfering signals from each area.

XPEEM results of both domains are shown in Figure 5e,f for the Fe 3p and Si 2p line, respectively. The Fe 3p line reveals the presence of iron in both domains of type α and β. The iron concentration in type β is lower than in type α. Moreover, the Si 2p line of domains of both types energetically overlaps. This indicates that silicon is bound in both domains in the same chemical surrounding.

The comprehensive results of the LEEM-IV and XPEEM data suggest the presence of iron in domain β. Originally, iron was present in domain α only. The iron content observed in domain β after oxidation must therefore result from iron migration from domain α. After leaving domain α, the migrating iron binds to the silicon atoms in domain β and again forms small agglomerates of ML-FeSiO_x_. For FeO coverages smaller than 50%, these agglomerates are not visible in form of a front, but in small particles of 50 nm size in average closely surrounding domain α. The LEEM-IV curve of domain α is identical with the LEEM-IV curve of ML-FeSiO_x_, as found in complete layers (Figure 2c). Thus, the formation of incomplete layers can directly be transferred to complete layers. The loss of iron as found in domain α directly shows that the iron concentration in FeSiO_x_ is smaller than in FeO. From the study of incomplete FeO films with silicon amounts necessary to form ML SiO_2_, it can be concluded that only two iron atoms per silica unit cell are present instead of three.

### 4.4 Incomplete Films BL-FeSiO_x_

**Figure 6:**
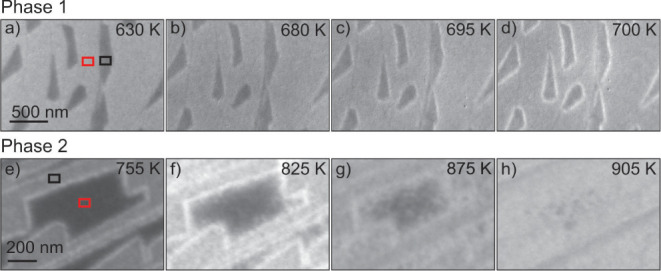
LEEM images during the oxidation of incompletely closed BL-FeSiO_x_ in 1.0·10^–6^ mbar O_2_. (a–d) Phase 1. (e–h) Phase 2. The temperature is increased by 0.5 K/s.

Similar to the previous part, incomplete layers of BL-FeSiO_x_ are prepared by using an unclosed film of bilayer-thick FeO and the silicon amount necessary to form a complete layer of SiO_2_. The two domains are assigned as type α (Si/BL FeO/Ru(0001)) and type β (Si/Ru(0001)). The oxidation is followed in LEEM. Similar to the case of incomplete layers of ML-FeSiO_x_, iron migrates from domain α and forms ML-FeSiO_x_ agglomerates with silicon dioxide in domain β. In contrast, the iron migration takes place in temperature-dependent phases: phase 1 with *T* < 800 K and phase 2 with *T* > 800 K. LEEM results during oxidation of the individual phases are given in Figures 6a–h for phase 1 and phase 2, respectively. Domains α and β are indicated by a black and red square, respectively. Different electron energies in Figures 6a–h lead to a contrast change between phase 1 and phase 2.

**Figure 7:**
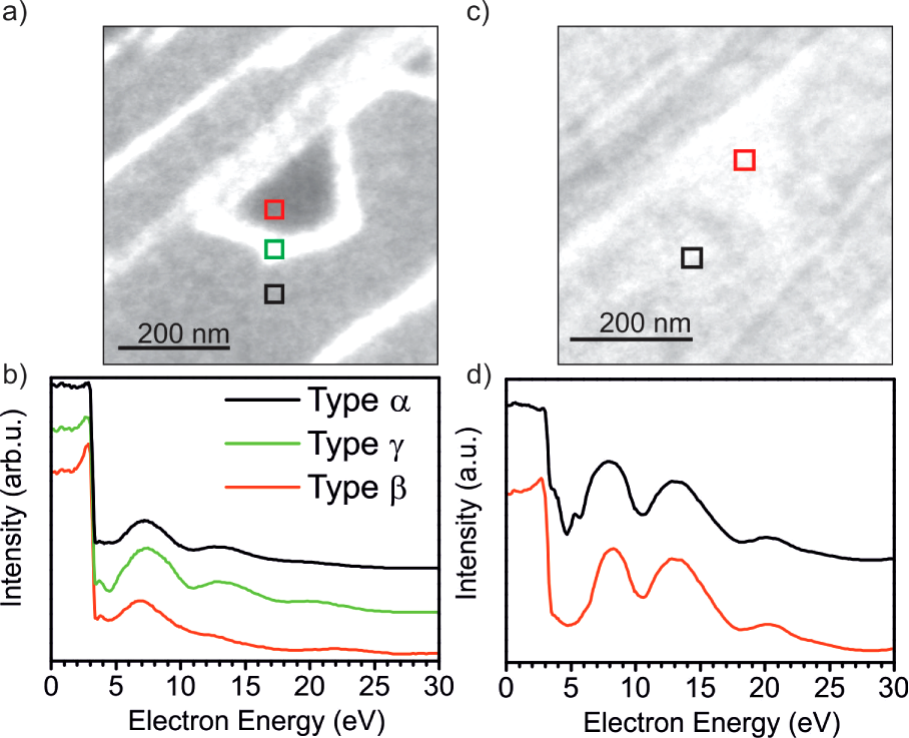
LEEM and LEEM-IV curves of (a,b) phase 1 oxidation (<800 K) and (c,d) phase 2 oxidation (>800 K) in 1.0·10^–6^ mbar O_2_. (a) LEEM image after oxidation at 800 K taken at 14 eV. (b) LEEM-IV curves of the (a) indicated domains. (c) LEEM image after oxidation at 900 K taken at 20 eV. (d) LEEM-IV curves of the (c) indicated domains.

In Figure 6a, the film is already oxidized at 630 K, and the initial contrast between domains of type α (dark) and type β (gray) is still present. The film oxidized at 630 K can be identified as phase 1. At 660 K, a bright rim forms surrounding domains of type α which will be addressed as domains of type γ from now on. The intensity of type γ is very low in the beginning but gains intensity up to 800 K until its reflectivity is higher than that of domain α (see Figure 6d at 700 K). The corresponding LEEM-IV curves after oxidation at 800 K are shown in Figure 7b. Domain α and domain γ share similar characteristics: peaks at 8 eV, 13 eV, and 21.1 eV. These peaks resemble the form of the peaks in the fingerprint of ML-FeSiO_x_ (see Figure 2c); however, they are shifted by 0.5 eV as in BL-FeSiO_x_ (see Figure 2d). Moreover, type γ contains a dip at 4.5 eV, while type α contains no such a dip. The single dip equals the one in ML-FeSiO_x_ if oxidized at 800 K (see Figure 2c) and thus suggests that domain γ is ML-FeSiO_x_. The iron atoms of type γ must result from migrating iron atoms of type α. The LEEM-IV curve of type β is relatively featureless with only one peak at 7 eV and resembles the fingerprint of a disordered SiO_2_ monolayer.

The second oxidation phase can be observed at temperatures above 800 K. LEEM images during oxidation are shown in Figures 6e–h. In this series, the FeO coverage is with 87% relatively high. At the chosen kinetic energy of electrons, domain α is bright, while domain β is dark. Domain γ is nicely visible containing a relatively sharp border. With increasing temperature, the border of domain γ becomes fuzzy and a front starts to grow toward the center of domain β (Figure 6f,g). The front is very inhomogeneous and appears as an agglomeration of many small domains of approximately 50 nm size. In fact, if the FeO coverage is lower than 50%, not a front, but separated agglomerates are accumulating in close surrounding of domains of type α. If domains of type β are very small compared to domains of type α, a complete transformation of domain β is possible. At the same time, the reflectivity of domains of type α increases until the same reflectivity as in the rim is present. Also, domain β assimilates to the reflectivity of domain α (Figure 6h).

The LEEM-IV characteristics for this case are depicted in Figure 7d. The curves of domain α and β are alike. Both curves contain peaks at 13 and 20.4 eV, similar peaks at 7.9 eV (type α) or 8.1 eV (type β). Domain α contains a sharp double dip at 4.7 and 5.7 eV and resembles the curve of ML-FeSiO_x_ in complete (Figure 2c) and incomplete layers based on a monolayer of FeO (Figure 5c). Thus, domain α is transformed from Si/BL FeO/Ru into ML-FeSiO_x_. Domain β contains only a very broad dip at 4.7 eV. As discussed before, the evolution from a single-dip to a double-dip shape in this energy regime testifies the structural quality of the FeSiO_x_ film. Thus, the film in domain β appears to have similarities with the ML-FeSiO_x_ film oxidized only at 800 K instead of 1000 K (Figure 2c).

**Figure 8:**
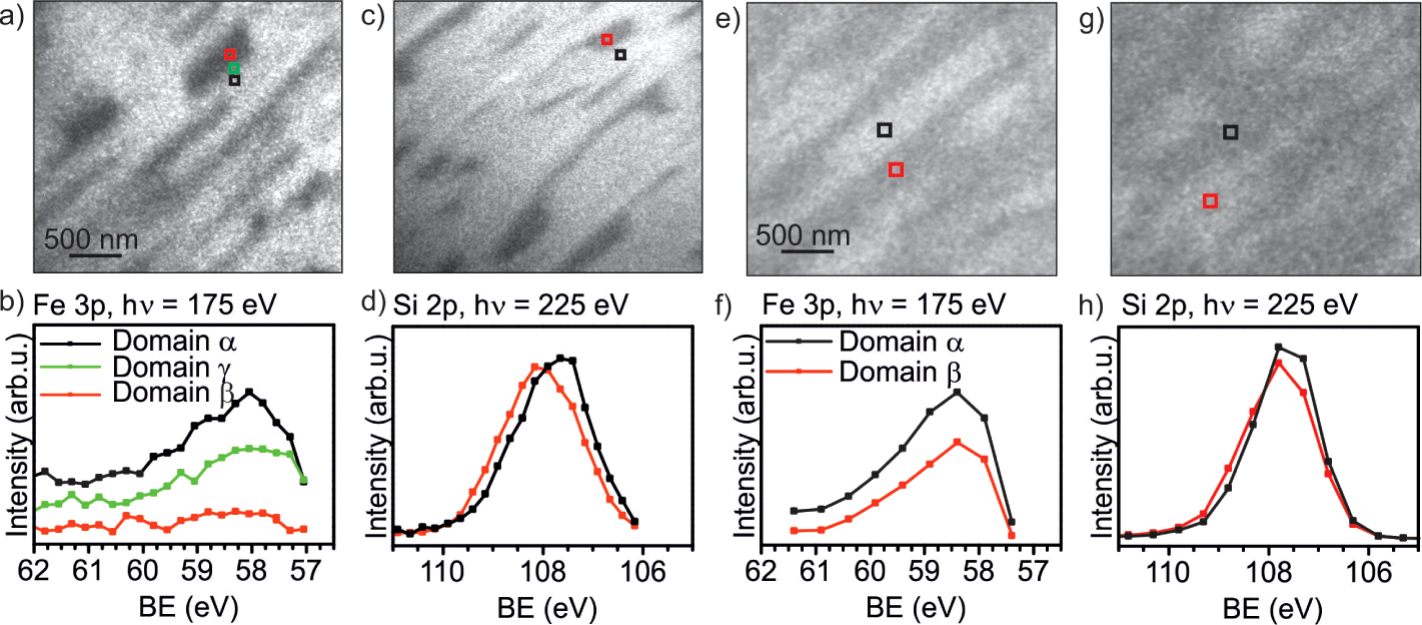
XPEEM results for phase 1 oxidation (<800 K) (a–d) and phase 2 oxidation (>800 K) in 1.0·10^–6^ mbar O_2_ (e–h). Phase 1: XPS Fe 3p with hν = 175 eV. (a) XPEEM image at 58.8 eV binding energy and (b) XPEEM Fe 3p line scan. Si 2p, hν = 225 eV. (c) Si 2p XPEEM image at 107.4 eV and (d) XPEEM scan at the indicated areas. (e) XPEEM image at 57.9 eV binding energy and (f) XPEEM Fe 3p line scan. Si 2p, hν = 225 eV. (g) Si 2p XPEEM image at 108.8 eV and (h) XPEEM scan at the indicated areas. Between the Fe 3p and Si 2p line, the sample position has been shifted.

XPEEM results of the Fe 3p and Si 2p lines are shown in Figures 8a–d, respectively. The Fe 3p line shows the presence of iron in the domain of type α and with a lower amount in type γ. In domains of type β, no iron is present. Using the Si 2p line, it is not possible to resolve domains of type γ; therefore, only domains of type α and β are compared. Both types indicate the presence of completely oxidized silicon with the Si^4+^ component. However, both lines are shifted by 0.8 eV against each other, presumably due to different chemical surroundings of either Si-O-Fe (domain α) or Si-O-Ru (domain β).

The XPEEM results of the Fe 3p and Si 2p line of domain α and β are depicted in Figures 8e–h, respectively. Both domain α and domain β contain iron. The iron content in domain α exceeds the one of domain β (Figure 8f). Moreover, the Si 2p line in domains of type α and β is nearly not or only slightly shifted (Figure 8h). This indicates a comparable chemical surrounding in both cases.

To summarize, two separate oxidation phases take place in incomplete layers of BL-FeSiO_x_. In the first oxidation phase (<800 K), iron migrates out of domains of type α (Si/BL FeO/Ru) toward domain β (Si/Ru) and binds directly at the border to the available silicon dioxide. Both XPEEM and LEEM-IV results suggest the iron silicate nature of the forming rim (type γ) containing presumably only one iron oxide layer. Since the temperature range of iron migration equals the one found in incomplete layers of ML-FeSiO_x_, the migrating iron correlates supposedly with the reduction of the iron concentration per iron oxide layer from three iron atoms per silica unit cell to only two. The migration of iron is limited in this phase to the rim. The rest of domain β contains no iron, and the fingerprint is similar to a disordered monolayer of SiO_2_.

For temperatures above 800 K, the rim dissolves and agglomerates of small particles with 50 nm size migrate toward domains of type β. This process is called the second oxidation phase. In case the FeO coverage exceeds 70%, agglomerates combine to a front which covers domains of type β completely. XPEEM results indicate that the agglomerates contain iron, and the LEEM-IV curves reveal a similar composition as complete layers of ML-FeSiO_x_, despite too low oxidation temperature. Thus, the LEEM-IV curve reveals the discontinuity of agglomerates. The original domains of type α (Si/BL FeO/Ru) exhibit the same LEEM-IV curve as complete and incomplete layers of ML-FeSiO_x_. This shows that the migrating iron in phase 2 stems from the dissolution of the second iron oxide layer. Since the Si-O-Fe bond is presumably strong and still present in the final films, the iron found in domain β correlates to the iron atoms of the iron oxide layer in contact to the Ru(0001) substrate.

The different manifestation of the migrating iron atoms in incomplete layers of ML-FeSiO_x_ and BL-FeSiO_x_ correlates most likely to the properties of the second iron oxide layer in BL-FeSiO_x_ domains. Dangling bonds at the border of these islands in the second iron oxide layer may trap the iron atoms, hindering their migration. Once the dissolution temperature of this second iron oxide layer is reached, the migrating iron atoms are released and steadily diffuse toward the center of domain β.

The same LEEM-IV curve of ML-FeSiO_x_ is found in complete and incomplete layers, both in islands of monolayer and bilayer-thick FeO basis layers. Moreover, iron atoms leaving these domains combine with silicon available in the initially iron-free areas to agglomerates with the ML-FeSiO_x_ characteristics. This suggests that the structure and configuration of ML-FeSiO_x_ are the energetically most stable one, even preferred to iron-free ML SiO_2_. While incomplete layers of BL-FeSiO_x_ transform into ML-FeSiO_x_, this is not the case in complete layers of BL-FeSiO_x_. This is evident by the different LEEM-IV curves and the higher Fe-O-Fe component detectable in the O 1s line. The reason is most likely that unbound iron is not possible to leave. The reason is most likely that iron cannot escape in such a large amount by diffusion to the Ru(0001) substrate and also not by desorption due to the silica layer on top. In complete layers of BL-FeSiO_x_, a (3 × 3) structure is found additionally to the LEED pattern in ML-FeSiO_x_. A possible reason might either be that only in every second silica layer, the iron concentration is reduced from three iron atoms per silica unit cell to two or alternatively, migrating iron atoms position between the silica layer and iron oxide layer and thus lead to a corrugation of the silica layer.

### 4.5 Iron Germanate Films

**Figure 9:**
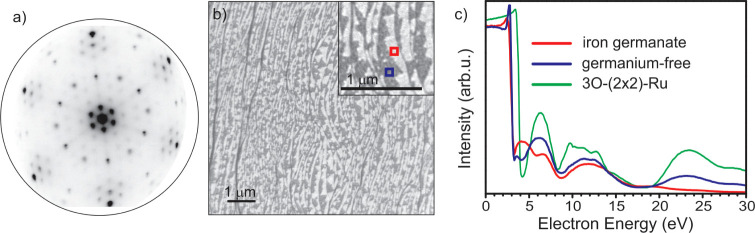
LEED and LEEM of an incomplete layer of iron germanate prepared by the stepwise recipe. The film is oxidized in 1.0·10^–6^ mbar at 720 K. (a) LEED pattern at 72 eV, measured after cooling down to RT. (b) LEEM image at 20 eV. (c) LEEM-IV curves taken in the areas indicated in (b). The LEEM-IV curve of an oxygen covered 3O-(2 × 2)-Ru(0001) surface is added for comparison reasons.

In this part, we present the formation of iron germanate films using the stepwise preparation (i.e., Fe deposition on an ordered ML GeO_2_). The initial GeO_2_ layer is incomplete, leaving some areas of uncovered ruthenium substrate. A complete layer of iron is deposited as a second step, i.e., covering both clean Ru and the germania film. We then expect two domains, one containing GeO_2_ + Fe/Ru (domain α) and the other Fe/Ru (domain β). Subsequently, the film is oxidized in 1.0·10^–6^ mbar. The size of domain α does not change during the oxidation process. Thus, a migration of germanium is not likely. The corresponding LEED and LEEM images after oxidation at 720 K are shown in Figure 9a,b. In the LEEM image (Figure 9b), domains α and β are indicated in red and violet, respectively. In Figure 9c, the corresponding LEEM-IV curves are shown.

The LEED pattern (Figure 9a) shows the superposition of multiple LEED spots: A Moiré pattern with “6 on 7” pattern, 30°-rotated spots, spots close to the (1 × 1) ruthenium positions, and a (2 × 2) pattern. The (2 × 2) pattern is found to disappear for annealing temperatures above 670 K, while the other spots enhance their intensity. However, during cooling, the (2 × 2) spots reappear. This behavior is known for oxygen on Ru(0001) and is a result of the reversible disorder of the oxygen atoms of 3O/Ru^6^. Therefore, holes of the film down to the 3O-(2 × 2)-Ru(0001) substrate can be observed. In Figure 9c, the LEEM-IV curve of 3O-(2 × 2)-Ru(0001) is given and compared with the LEEM-IV spectra in the individual domains. While the curve of domain α (iron germanate) is clearly different, especially by the presence of the peak at 4 eV, the LEEM-IV curve of domain β (germania-free) shows similarities with the LEEM-IV curve of oxygen-covered Ru (peaks at 6.4 eV, 11.2–13.0 eV, and 23.2 eV). Nevertheless, the MEM-LEEM border is clearly lower than the one of purely oxygen-covered ruthenium, which indicates a smaller surface dipole. Moreover, the peak between 10.0 and 12.0 eV is broader for 3O-(2 × 2)-Ru(0001) than the measured peak and contains a higher intensity at 10.0 eV than at 12.0 eV. These findings suggest that in domain β, iron oxide is still present after oxidation at 720 K. However, the (2 × 2) spots are very intense. Therefore, the iron amount in this domain is very low.

Iron germanate is only found in domain α. The LEED pattern of these domains is a superposition of the Moiré pattern, the 30°-rotated spots, and those close to the (1 × 1) substrate spots. All spots of this pattern are already present at 620 K. However, the spots are blurry, and their intensity is low. With increasing temperature up to 860 K, the spot intensity increases and the spot width decreases. At 860 K, the LEED pattern is optimal and the spots are sharpest. For higher temperatures, the spots rotated by 30° diminish.

**Figure 10:**
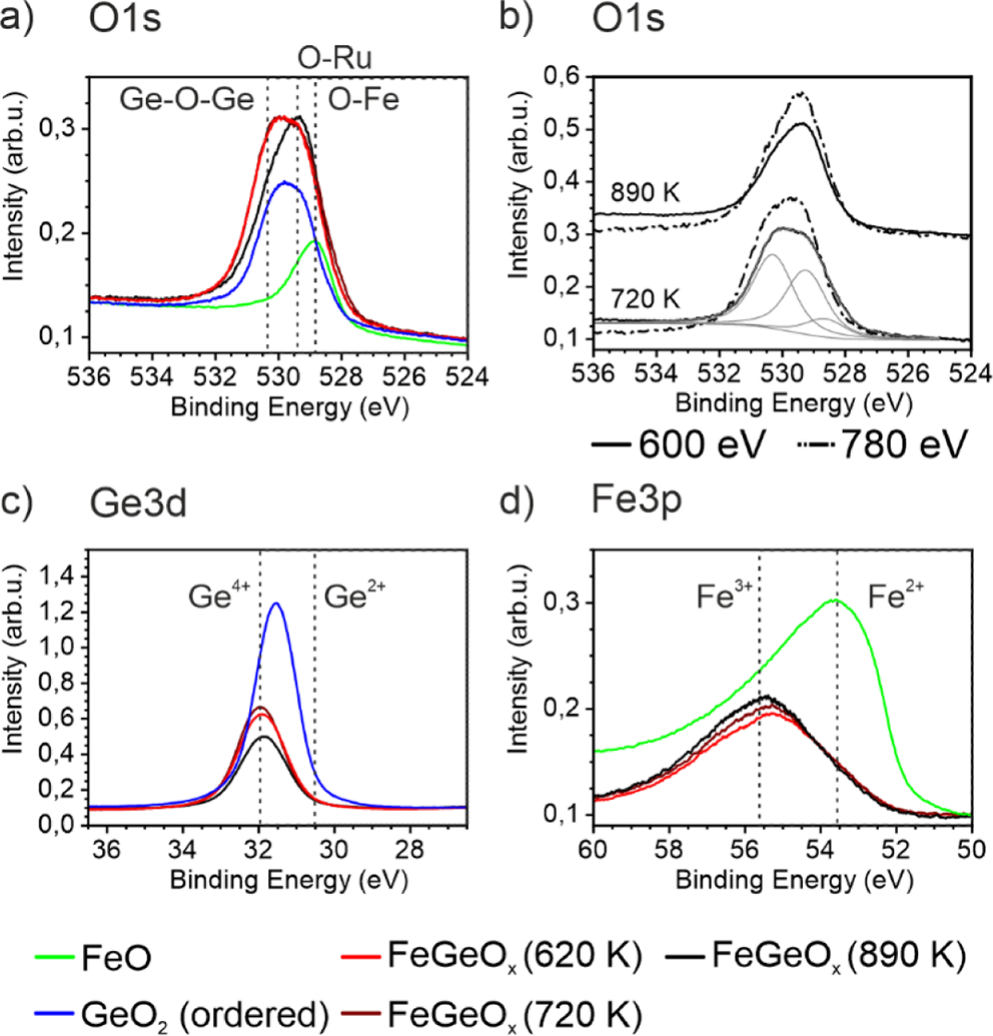
XPS analysis of iron germanate produced with the stepwise recipe. The lines of iron germanate oxidized in 1.0·10^–6^ mbar at 620 K, 720 K, and 890 K, and for comparison, those of the pure ordered ML of GeO_2_ and of the pure FeO monolayer are shown. (a) O 1s line, hν = 600 eV. (b) O 1s line of iron germanate with varying photon energy and thus varying probing depth. In light gray, the fit of the O 1s line after 720 K is indicated. (c) Ge 3d line, hν = 100 eV. (d) Fe 3p line, hν = 175 eV.

The XPS spectra of the O 1s, Ge 3d, and Fe 3p lines after oxidation at 620 K, 720 K, and 890 K are given in Figures 10a–d, respectively. The XPS lines for a pristine crystalline GeO_2_ monolayer and a pure FeO monolayer are added. The pure germania monolayer consists of a 6-fold ring system, including Ge-O-Ge bonds parallel and O-Ru bonds perpendicular to the surface.^10^ In the O 1s line (Figure 10a), these components are found at 529.3 eV (O-Ru bond) and 530.2 eV (Ge-O-Ge bond). Germanium is in the Ge^4+^ state (Figure 10c). The pristine FeO monolayer creates one component at 528.9 eV in the O 1s line and thus overlaps with the O-Ru component at 529.3 eV. Iron is present in the Fe^2+^ state, as shown in Figure 10d.

The XPS lines of the iron germanate preparation represent an average over both domains α and β. Thus, in evaluating iron germanate, the component at 529.3 eV is overestimated due to the iron oxide layer in domain β. Iron and germanium are found completely oxidized after temperatures of 620 K. The contributions of the single components of the O 1s core level are shown in Figure S2. Germanium is found in the Ge^4+^ state (Figure 10c), while iron is oxidized in the Fe^3+^ and Fe^2+^ state (Figure 10d). The O 1s line is shown in Figure 10a. The O 1s line after heating to 720 K can be fitted either with two or three components, which can be correlated to Ge-O-Ge and Fe-O-Fe/O-Ru or Ge-O-Ge, Ge-O-Fe, and Fe-O-Fe/ O-Ru, respectively. It turns out that the fit with two components shows equal intensity in the Ge-O-Ge and Fe-O-Fe line. This would suggest that the structure is not dispersed into two layers, but germania and iron oxide are intermixed, so that none of the individual elements are damped. Moreover, the two components would exclude Ge-O-Fe bonds. In Figure 10b, the fit with three components is shown. For the oxidation at 620 and 720 K, no significant change is found in any of the XPS lines. This indicates that iron germanate is completely oxidized at 620 K, and the oxidation states are not changed for higher temperatures. The LEED spectra indicated that it is mainly its structure that improves up to 860 K. As discussed before, the intensity of the spots rotated by 30° of the iron germanate LEED pattern decreases at 860 K. In the O 1s line, the Ge-O-Ge component decreases strongly in the same temperature range, and also, the Ge 3d intensity decreases. In contrast, the intensity in the Fe 3p line increases. These results indicate that germanium evaporates at temperatures above 860 K, while the iron oxide layer remains on the substrate. Moreover, the depth profile of the O 1s line for photon energies of hν = 600 and 780 eV displayed in Figure 10b shows no significant difference between surface-sensitive (hν = 600 eV) and less surface-sensitive measurements (hν = 780 eV), in contrast to iron silicate shown in Figure 3b. Most likely, the very large component at 529.3 eV (Fe-O-Fe and O-Ru bonds) is due to the O-Ru and Fe-O-Fe bonds in domain β.

Iron germanate has been found to have very similar LEED and XPS characteristics as iron silicate. Both give rise to a Moiré pattern, spots rotated by 30°, and the iron-related spots close to the (1 × 1) ruthenium spots. Moreover, the oxidation states of silicon (Si^4+^) and germanium (Ge^4+^), as well as the oxidation states of iron (Fe^3+^ and Fe^2+^), are comparable. Thus, a similar structure for both systems is expected. This also concerns the presence of three components in the O 1s line in iron germanate.

Regarding the LEED structure, it is found that the Moiré pattern differs for both films: “6 on 7” in iron germanate and “8 on 9” in iron silicate. Thus, six iron atoms overlap commensurably with seven ruthenium atoms in iron germanate and eight iron atoms with nine ruthenium atoms in iron silicate. The next neighbor distance of Ru atoms in Ru(0001) is 2.706 Å. From this, the Fe-Fe distance can be determined as (3.16 ± 0.03) Å and (3.04 ± 0.03) Å in iron germanate and iron silicate, respectively. The Ge-O-Ge and Si-O-Si bond lengths correlate with the Fe-Fe distance and are thus determined as (3.16 ± 0.03) Å and (3.04 ± 0.03) Å, respectively. As discussed in Subsection 2^2^, the Si-O-Si bond distance in iron silicate agrees within the error bars with the Si-O-Si bond length in unstrained silicates.

The most frequent Ge-O distance in unstrained germanates is 1.73 Å.^36^ The mean value of Ge-O-Ge bond angles in unstrained germanates is 133°.^37^ From these values, the intermediate value of 3.17 Å for the Ge-O-Ge bond length is derived. In fact, this value equals, within the error, our determined Ge-O-Ge length in iron germanate of (3.16 ± 0.03) Å. This shows that both the Ge-O-Ge and Si-O-Si bonds in iron germanate and iron silicate are relaxed, while the Fe-Fe bonds differ in both films. This strongly suggests that it is the germania or silica layer that determines the Fe-Fe distance and not, for instance, the Ru substrate.

### 4.6 DFT Modeling

**Figure 11:**
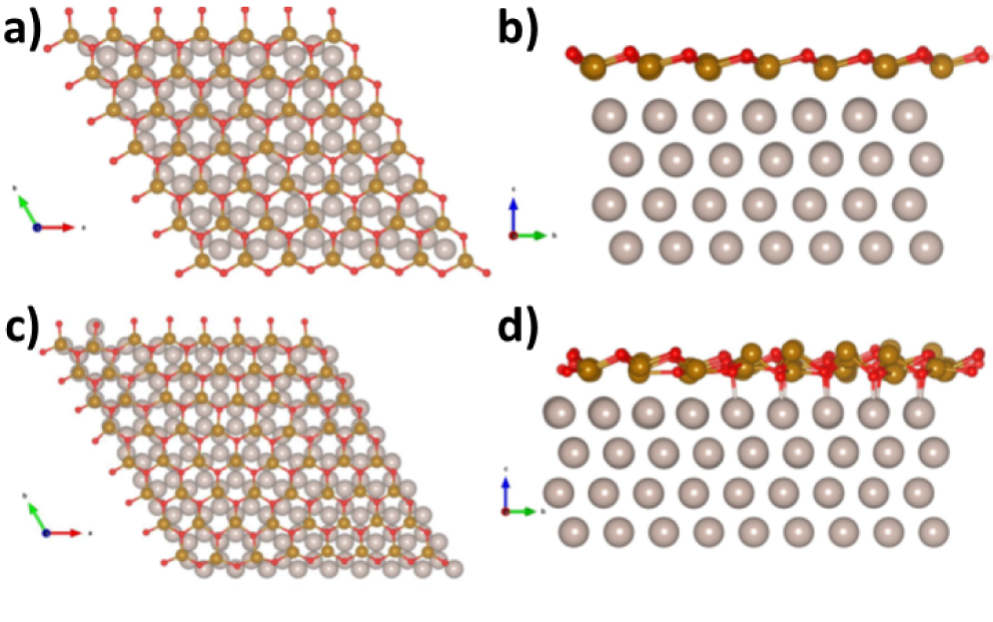
Top view (a) and side view (b) of FeO(6 × 6) on Ru(7 × 7). Top view (c) and side view (d) of FeO(8 × 8) on Ru(9 × 9).

We start the analysis of the computational results from the modeling of the FeO films on Ru(0001) (Figure 11). A monolayer of FeO is formed, displaying hexagonal cages. The most favorable stacking envisages the Fe ions in contact with the Ru substrate and the O ions pointing upward, in agreement with previous computational results.^38^ The iron-iron mean distance depends remarkably on the lattice matching with the Ru substrate (Table 1): on the FeO(6 × 6)/Ru(7 × 7) coincidence, it is 3.12 Å, in good agreement with the experimental value of 3.16 Å (Section 4.1). This value decreases to 3.00 Å (3.04 Å in the experiment) in the FeO(8 × 8)/Ru(9 × 9) coincidence, the one observed for the iron silicate films. Notably, the main physical properties of the FeO films are quite similar for both structures: a moderate increase in work function is observed with respect to bare Ru (5.13 eV with the computational setup adopted here), while the Bader charges indicate a partial oxidation of the Fe atoms in interaction with Ru (for comparison, the Bader charges of iron in bulk FeO and Fe_2_O_3_ are 1.4 |e| and 1.9 |e|, respectively^39^). The structure of the (6 × 6)/(7 × 7) reconstruction displays a more regular vertical arrangement of the FeO film (Figure 11b) with respect to (8 × 8)/(9 × 9) (Figure 11d).

**Table 1:** Main Calculated Properties of FeO, FeSiO_x_, and GeSiO_x_ Films on Ru(0001)^a^

	<d> (Fe-Fe), Å	WF, eV	<BE> (Fe 3p), eV	Min-Max q(Fe), |e|
FeO(6 × 6)/Ru(7 × 7)	3.12	5.51	53.8	0.86–1.17
FeO(8 × 8)/Ru(9 × 9)	3.00	5.59	54.0	0.86–1.17
FeSiO_x_ complete1	2.99	6.30	54.1	0.92–1.58
FeSiO_x_ complete2	2.86	6.26	54.0	0.89–1.53
FeSiO_x_ 1:1	2.94	5.97	54.2	0.95–1.56
FeGeO_x_ complete1	3.05	6.69	54.1	1.00–1.54
FeGeO_x_ complete2	3.06	6.03	54.1	1.11–1.45
FeGeO_x_ 1:1	2.93	6.10	54.0	0.51–1.52

a Mean Fe-Fe distance, work function, mean binding energy of Fe(3p) core electrons, and minimum and maximum Bader charges of Fe atoms.

**Figure 12:**
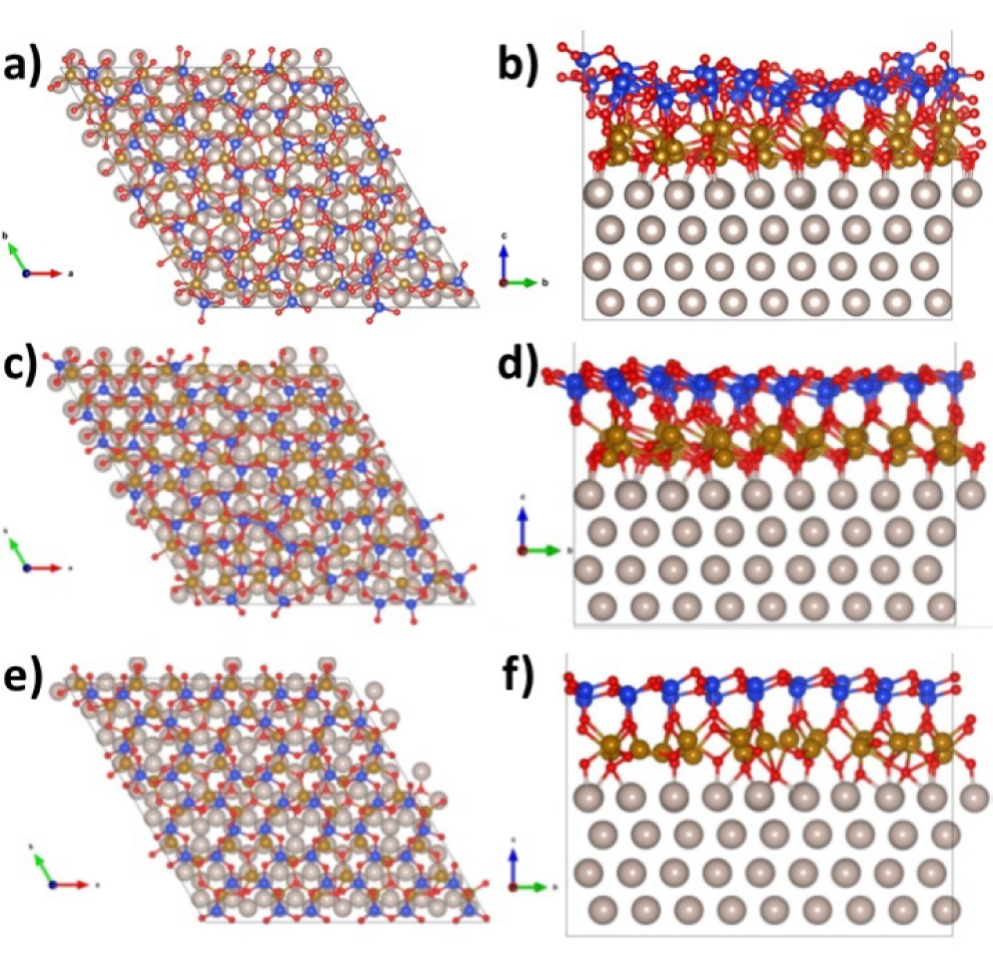
Top view (a) and side view (b) of FeSiO_x_/Ru, model “complete1”. Top view (c) and side view (d) of FeSiO_x_/Ru, model “complete2”. Top view (e) and side view (f) of FeSiO_x_/Ru, model “1:1”.

**Figure 13:**
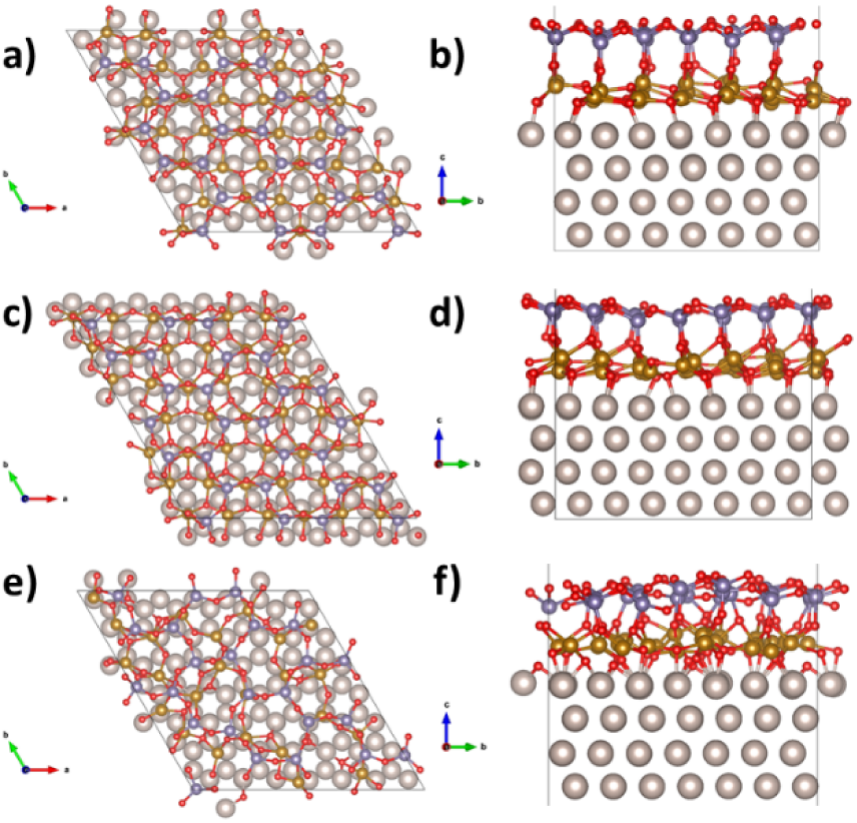
Top view (a) and side view (b) of FeGeO_x_/Ru, model “complete1”. Top view (c) and side view (d) of FeGeO_x_/Ru, model “complete2”. Top view (e) and side view (f) of FeGeO_x_/Ru, model “1:1”.

Next, we discuss the models derived for iron silicate and germanate on Ru(0001). Based on the experimental evidence presented in the previous sections, we use the (8 × 8)/(9 × 9) lattice coincidence for SiO_2_ and (6 × 6)/(7 × 7) for GeO_2_. In all structures, a reciprocal 30° rotation between FeO and SiO_2_ (or GeO_2_) is envisaged. In both cases, we consider two possibilities: i) the SiO_2_, or GeO_2_, films grow over a complete FeO layer (with stoichiometric rations of Fe/Si = 1.2 and Fe/Ge = 1.5) or ii) a migration of FeO toward clean Ru takes place during the growth, as discussed in Section 4.4, leading to structures displaying only two Fe atoms per (SiO_2_) unit cell (i.e., a 1:1 stoichiometric ratio between Fe and Si or Ge). For the case of the complete FeO monolayer, we consider two possible initial vertical stackings: Ru, Fe, O, O, Si(Ge), and O (model complete1) or Ru, O, Fe, O, Si(Ge), and O (model complete2). To generate the FeO-poor model, displaying an equal number of Fe and Si(Ge) atoms per supercell, we started from the structure of 50% iron-substituted SiO_2_ bilayer previously proposed by Włodarczyk et al.,^15^ applied a 30° rotation between the two layers, and adapted the resulting structure to a (9 × 9) (silica) or (7 × 7) (germania) Ru supercell; we refer to this type of interface as “1:1” model in the following. The relaxed structures are displayed in Figure 12 (iron silicate) and Figure 13 (iron germanate). The main properties of the films are reported in Table 1.

For iron silicate, we observe that the complete1 model (Figure 12a,b) displays a very disordered structure, with a remarkable rumpling of the silica top layer (a feature not observed in the experiments). The complete2 structure (Figure 12c,d), on the contrary, preserves a plausible morphology but is metastable with respect to complete1 by 0.14 eV per FeO stoichiometric unit. This fact indicates a poor match between FeO and silica, leading to a high structural disorder. It is also worth mentioning that the average Fe-Fe distance is very short in complete2 (2.86 Å), while it has a more plausible value in complete1 (2.99 Å) (Table 1). The 1:1 model (Figure 12e,f) displays a rather good degree of structural homogeneity and a Fe-Fe distance (2.94 Å) slightly shorter than the corresponding FeO/Ru interface (Table 1). In all three models, the Fe atoms undergo a remarkable oxidation with respect to FeO/Ru, as indicated by the increase in both the Fe (3p) binding energy and the Fe Bader charges (Table 1). In the case of the complete models, a very large increase in the work function with respect to bare Ru is observed (6.30 eV for complete1 and 6.26 eV for complete2 with respect to 5.13 eV for bare Ru). For the 1:1 model, the WF increase is less strong (5.97 eV, Table 1).

The case of iron germanate is quite different, in the sense that the complete models (Figures 13a–d) display a more ordered structure with respect to 1:1 (Figure 13e,f). Moreover, at variance from the iron silicate case, the complete2 model is more stable than complete1 by 1.74 eV per FeO stoichiometric unit. The average Fe-Fe distance is larger than in the case of silica for the complete models (3.05 Å for complete1 and 3.06 Å for complete2, Table 1), while it is remarkably shorter for 1:1 (2.93 Å). The most stable complete2 model displays a work function of 6.03 eV, 0.90 eV larger than bare Ru(0001). The average Fe(3p) binding energy is very close to what is observed for iron silicate (Table 1). The Fe Bader charges are also similar to the silicate case and span over a range which is compatible with the coexistence of iron species with +2 and +3 oxidation states. Complete1 has similar BE (Fe3p) and Q(Fe) to complete2 but displays a remarkably larger work function (6.69 eV). The 1:1 model, finally, displays work function and average BE(Fe3p) in quite close agreement with the complete FeO models, but some Fe ions have a very small Bader charge (the minimum value is 0.5 |e|).

**Figure 14:**
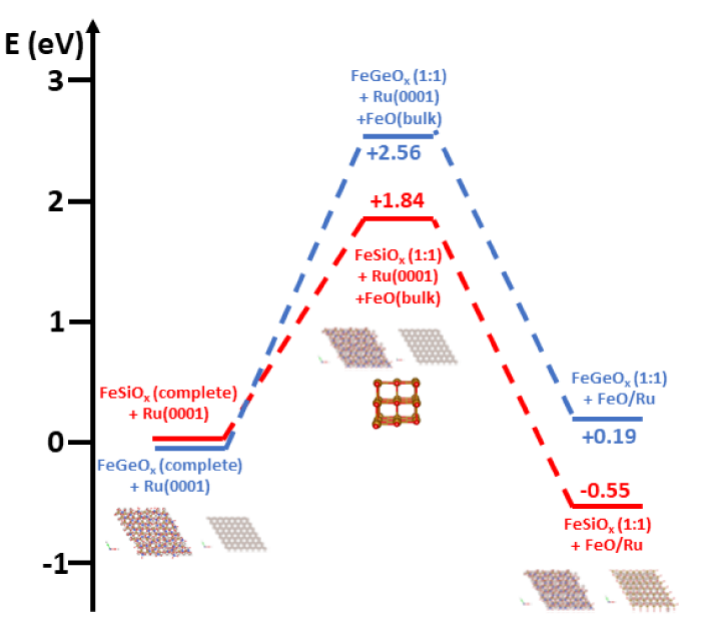
Two-step Born cycle leading from the “complete” model to the “1:1” model, envisaging (1) a loss of FeO from the complete model to form bulk FeO and (2) the deposition of FeO on ruthenium to form FeO/Ru. Units in eV/FeO stoichiometric unit.

Comparing the thermodynamic stability of complete1 and complete2 models is trivial, since the respective supercells contain the same number of atoms. As discussed above, this leads to the conclusion that complete1 is more stable than complete2 for iron silicate, while the reverse order of stability is obtained for iron germanate. However, assessing the relative stability of the FeO-poor 1:1 models with respect to the complete models is more complicated, due to the chemically unbalanced content of the supercells. To do this, we recur to a virtual two-step chemical reaction forming a Born cycle, as shown in Figure 14: the starting point, and thermodynamic reference, is the most stable complete-FeO model for either FeSiO_x_ or FeGeO_x_, plus the clean Ru(0001) surface, at the left end of Figure 14. Next, we evaluate the energy price involved with the loss of a given number of FeO stoichiometric unit, leading to the 1:1 model. To this end, we use bulk iron oxide as a thermodynamic reference for FeO (Figure 14, middle). Then, we virtually adsorb the FeO released from the complete model on clean Ru, forming the FeO/Ru overlayer (Figure 14, right end). It can be noticed that the overall process leading from the FeO-complete1 model to 1:1 model + FeO/Ru is exothermic by 0.55 eV/FeO unit for iron silicate. On the contrary, for FeGeO_x_, the reaction from complete2 to 1:1 is endothermic by 0.19 eV/FeO unit.

We thus conclude that, in analogy with the experimental observations described in experimental part, FeO migration to clean Ru during silicon oxidation could possibly take place, creating a structure with only two Fe atoms per SiO_2_ unit cell. Vice versa, iron germanate can grow on a complete FeO(6 × 6)/Ru(7 × 7) superstructure without loss of FeO. The most stable iron silicate (1:1) and iron germanate (complete2) model structures display Fe-Fe average distances, work function, average BE(Fe3p), and Bader charges in reasonable agreement with the experimental evidence. We must state, however, that BE(Fe3p) and Bader charges are quite similar for all the models and are thus of little help in identifying the correct structure. In this respect, the analysis of the thermodynamic stability is the strongest evidence to support our assignment. A direct comparison of the experimental LEED pattern of iron silicate and germanate with the calculated DFT structures is shown in Figure S3.

## 5 Conclusion

Our results show that iron silicate can be prepared on the basis of a monolayer and a bilayer of FeO. The preferred configuration is ML-FeSiO_x_, which consists of a monolayer of SiO_2_ bound to a single layer of iron oxide. This iron oxide layer is hexagonally arranged with only two iron atoms per unit cell. This scenario is fully supported by the theoretical calculations and thus provides clear evidence for the proposed structure. As a result, the number of iron atoms per silica unit cell is reduced during oxidation if prepared on the basis of an ML FeO layer (three iron atoms per silica unit cell). We followed the reduction of iron by preparing films with unclosed layers of either monolayer or bilayer-thick FeO islands. In case of monolayer FeO, iron diffuses from the iron-containing areas to the iron-free areas by forming ML-FeSiO_x_ with the available silicon dioxide. In case of bilayer FeO islands, first the number of iron atoms per silica unit cell is reduced. The migrating iron atoms together with the available silicon dioxide form a rim at the border of islands with ML-FeSiO_x_ characteristic, thus also with only two iron atoms per silica unit cell. For temperatures above 800 K, the second iron oxide layer in contact to the Ru(0001) substrate dissolves and migrates into the initially iron-free areas. There again, ML-FeSiO_x_ agglomerates are formed. Next to the ML-FeSiO_x_ agglomerates in initially iron-free domains, silica monolayer domains are formed. The amount depends on the number of migrating iron atoms. In complete layers of BL-FeSiO_x_, both iron oxide layers remain, most likely due to missing escape possibilities of migrating iron atoms.

The comparative investigation of the temperature-dependent formation and the structures of ultrathin layers of iron silicate and iron germanate lead to an interesting conclusion based on the comparative theoretical study. In both cases, the films consist of a two-layered structure with a monolayer of silica or germania, respectively, on top of a monolayer of iron oxide. While in the case of FeSiO_x_, the most stable structure pertains to only two Fe atoms per unit cell, and for FeGeO_x_ a full layer of three Fe aoms persists in the most stable structure. This explains the observed iron oxide migration for the FeSiO_x_ system.

The Fe-Fe distance in these layers is mainly determined by the Si-O-Si and Ge-O-Ge bond length, respectively, and not by the substrate.

## Supporting Information Available

Contribution of O 1s components in FeSiO_x_ in dependence of the FeO thickness; contribution of O 1s components in FeGeO_x_ in dependence of the oxidation temperature; comparison of the FeSiO_x_ and FeGeO_x_ LEED pattern (PDF)
