# Supplementary material for: Growth and Structure of Ultrathin Iron Silicate and Iron Germanate Films
Source: J Phys Chem C Nanomater Interfaces. 2024 Oct 31;128(45):19423–35. doi: 10.1021/acs.jpcc.4c05601 (PMC11571205; doi:10.1021/acs.jpcc.4c05601)
Supplement: Supplementary file 1 — jp4c05601_si_001.pdf [file jp4c05601_si_001.pdf]

Supporting information

Growth and structure of ultrathin iron silicate and iron germanate films

Gina Peschel[1], Alexander Fuhrich[1], Dietrich Menzel[1], Mirali Jahangirzadeh Varjovi,[2] Sergio Tosoni,[2] and Hans-Joachim Freund[1]

[1]Fritz Haber Institute of the Max Planck Society, Faradayweg 4-6, D-14195 Berlin, Germany

[2] Department of Materials Science, University of Milano-Bicocca, Via Roberto Cozzi 55, 20125 Milano, Italy

Contribution of O1s components in $FeSiO_x$ in dependance of the FeO thickness

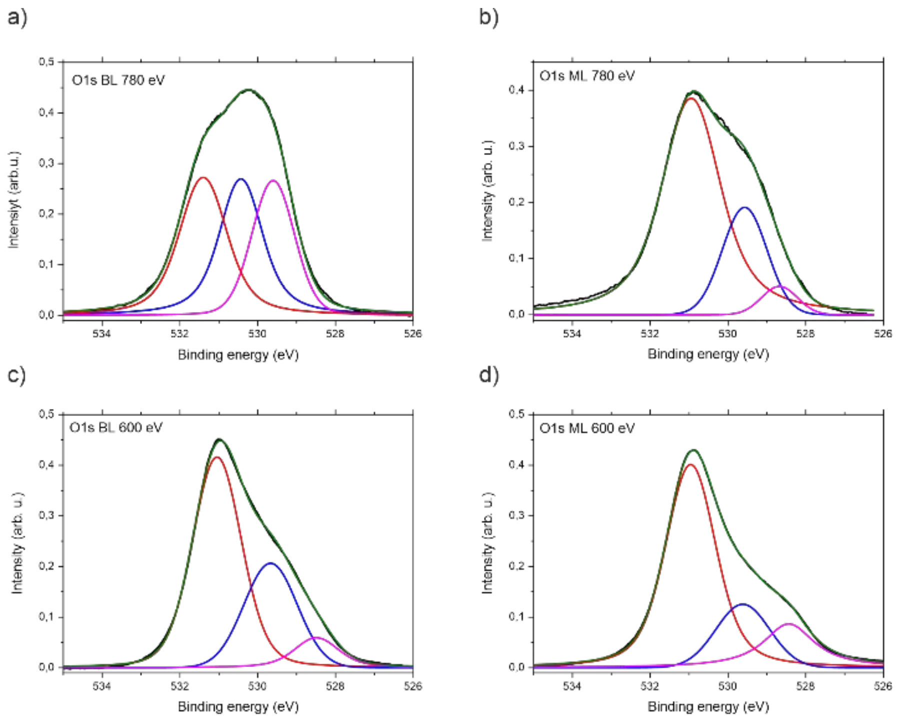

***Figure S 1**: Contribution of the O1s core level to BL-$FeSiO_x$ (a,c) and ML-$FeSiO_x$ (b,d). The O1s core level is fitted with three components assigned to Fe-O-Fe (violet), Si-O-Si (red) and Ru-O (blue). The change of the incident photon energy leads to a change of the kinetic energy of the photoelectrons. This leads to less surface sensitive*

information for higher photon energy. The XPS depth profile shows the layered structure of $FeSiO_x$ with the Si-O-Si component on the surface.

Contribution of O1s components in $FeGeO_x$ in dependance of the oxidation temperature

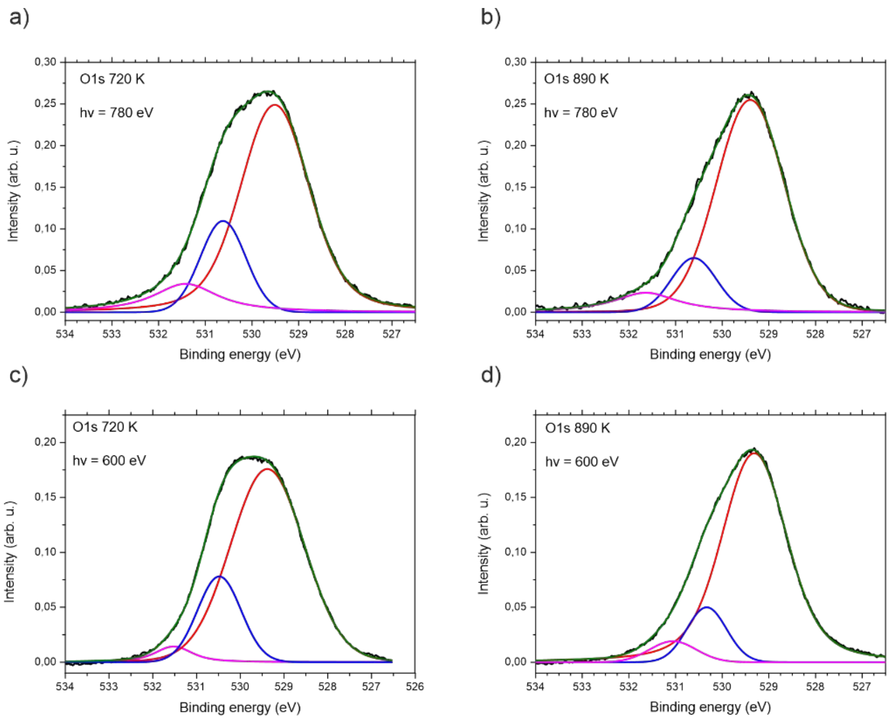

***Figure S 2**: Contribution of the O1s core level to $FeGeO_x$ for 720 K (a, c) and 890 K (b,d) annealing temperature in $1 \cdot 10^{-6}$ mbar O_2. The O1s core level is fitted with three components assigned to Fe-O-Fe (red), Ge-O-Ge (violet) and Ru-O (blue). The change of the incident photon energy leads to a change of the kinetic energy of the photoelectrons. This leads to less surface sensitive information for higher photon energy. The XPS depth profile shows the layered structure of $FeGeO_x$ with the Ge-O-Ge component on the surface.*

Comparison of $FeSiO_x$ and $FeGeO_x$ LEED pattern

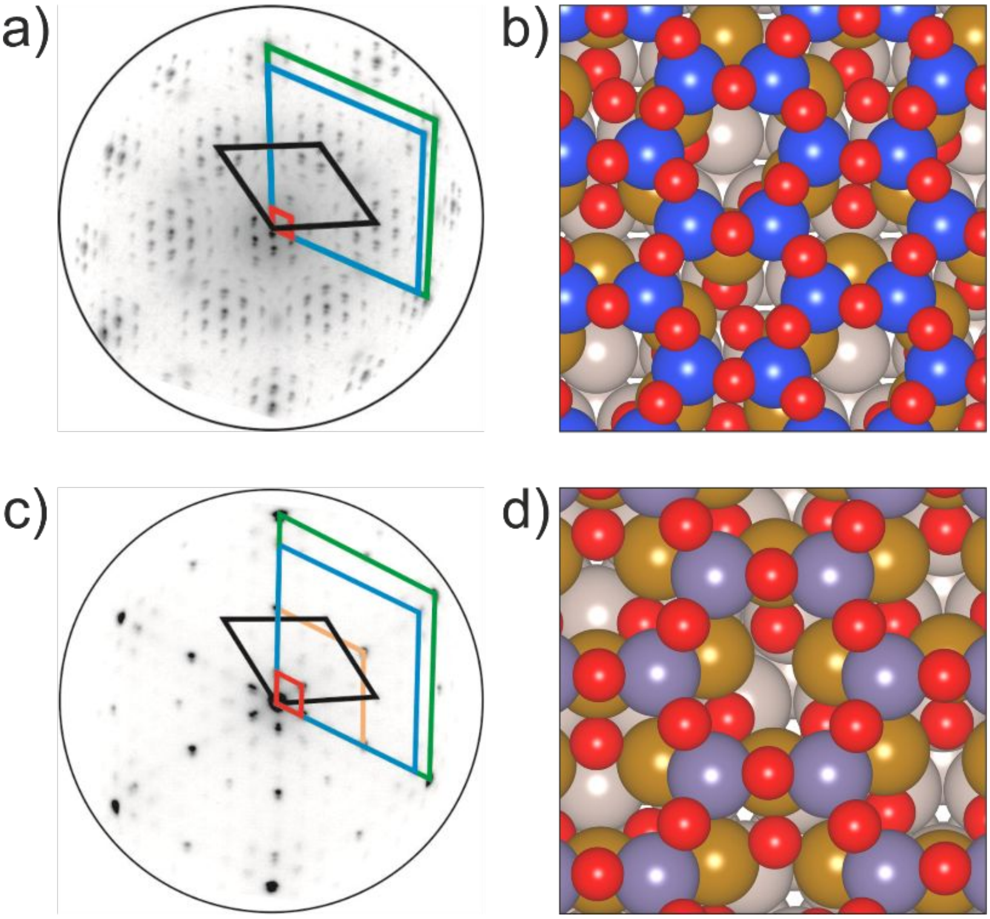

***Figure S 3**: Comparison of $FeSiO_x$ and $FeGeO_x$ LEED pattern (42 eV) with the calculated DFT structures. a) LEED pattern of $FeSiO_x$ with marked unit cells. The unit cell of the Ru(0001) support is labeled in green. The unit cell of the FeO Moiré pattern is shown in blue and the FeO unit cell in red. The Moiré pattern can be identified as "8 on 9". The black unit cell corresponds to SiO_2 (√3x√3) R30°. b) shows the calculated DFT structure of $FeSiO_x$ with 2 Fe atom per $FeSiO_x$ unit cell. c) LEED pattern of $FeGeO_x$ with marked unit cells. The unit cell of the Ru(0001) support is labeled in green. The FeO Moiré pattern is shown in blue and the FeO unit cell in red. The Moiré pattern can be identified as "6 on 7". The black unit cell corresponds to GeO_2 (√3x√3) R30° for the $FeGeO_x$ regions and the orange labeled unit cell corresponds to the 3O-(2x2)-Ru(0001) coverage in between regions with $FeGeO_x$. d) shows the calculated DFT structure of $FeGeO_x$ with 3 Fe atom per $FeGeO_x$ unit cell.*
